# Editorial: Introducing the Intercontinental NMR Seminar ICONS2020

**DOI:** 10.1007/s00723-020-01268-0

**Published:** 2020-10-12

**Authors:** Daniel Abergel, Gerd Buntkowsky, Konstantin L. Ivanov, Perunthiruthy K. Madhu

**Affiliations:** 1 Laboratoire des Biomolécules, Département de chimie, École normale supérieure, PSL University, Sorbonne Université, CNRS, 75005 Paris, France; 2 Institute of Inorganic and Physical Chemistry, Technical University of Darmstadt, D-64287 Darmstadt, Germany; 3 International Tomography Center SB RAS, Novosibirsk, 630090 Russia; 4 TIFR Centre for Interdisciplinary Sciences, Tata Institute of Fundamental Research Hyderabad, 36/P Gopanpally Village, Ranga Reddy District, Hyderabad, 500107 India

**ICONS2020,** the **I**nter**co**ntinental **N**MR **S**eminar, organized during August 26–28, 2020, was the first of a new on-line magnetic resonance conference series. It was an off-shoot of the weekly Intercontinental NMR Seminar Series that started on April 8, 2020. This seminar series enables communication and dissemination of research ideas among the magnetic research community especially in the times of the COVID-19 pandemic. The seminar and ICONS series cover various aspects of magnetic resonance, both electron and nuclear. The seminar series also gives early-stage researchers an opportunity to give seminar talks and interact with colleagues from different countries. The success of the seminar series prompted us to introduce a new conference to highlight the recent research contributions from some of the leading groups in magnetic resonance. ICONS2020 attracted registrations from nearly 550 people spanning 35 countries (in the spirit of the meeting, covering 5 continents). The meeting talks were broadcast across the Zoom and YouTube platforms. The average combined attendance was around 180 with the peak touching 250. The meeting covered a wide range of topics including methods in solid-state NMR, intrinsically disordered proteins (IDPs) and ^13^C detection, relaxation studies, exploiting residual anisotropy in solution-state NMR, NV centers in diamonds, theoretical modeling of dynamics in macromolecules, using probes such as ^19^F to probe long-range contacts in molecules under fast MAS and DNP conditions, application of solid-state NMR on MOF’s, DNP at higher magnetic fields and MAS frequencies, status and future of dissolution DNP, observations of subtle scalar couplings in fullerene systems alone and trapped with ^3^He, and scientific amusements.

Geoffrey Bodenhausen, ENS Paris, introduced the dissolution DNP technique, which provides enormous signal enhancements. Speaking about DNP methodology and applications, he mentioned not only the strengths of the technique but also about its weaknesses—a part often omitted in papers and conference talks. The bullet-DNP method was then proposed as a possible remedy and solution to the currently existing problems (faster polarization and transfer, less dilution and relaxation losses).

Vipin Agarwal, TIFR Hyderabad, highlighted the use of carefully designed pulse schemes in extracting ^1^H–^1^H distances in solid-state NMR under higher MAS frequencies. The talk focused on the design of SERP recoupling method and some of its variants along with a newer scheme based on C-symmetry.

Antonino Polimeno, University of Padova, presented a generalized master equation of the dynamics of a flexible molecule in solution represented by an all-atom model. This exact stochastic model can then be tuned to the required level of description using different choices of internal variables and levels of description of the solvent. The modelling protocol was presented for a semi-flexible model approximation, leading to the computation of NMR correlation functions and relaxation times.

Tatyana Polenova, University of Delaware, highlighted recent results on the methodology and application of ^19^F as NMR label in biological macromolecules and biomolecular assembles and discussed the importance of fast MAS spinning frequencies for the accurate determination of interfluorine distances and demonstrated the advantages of ^19^F DNP enhanced MAS NMR in the characterization of HIV-1 capsid protein ensembles.

Dieter Suter, University of Dortmund, introduced the concept of NMR experiments without RF-excitation. The way to run such experiments is to modulate electron-nuclear hyperfine couplings by applying microwave pulses to electron spins. A suitable test system is given by the negatively charged nitrogen-vacancy centers (NV^–^ centers) in diamonds, with optically induced spin hyperpolarization and sensitive optical readout of the spin state.

Yusuke Nishiyama, RIKEN, showed the design strategies of pulse methods to yield high-efficiency artefact-free triple-quantum ^1^H–^1^H correlation spectra, focusing more on MAS at 60 kHz. He also highlighted probing hydrogen-bonding structures in molecules combining microED and solid-state NMR.

Eike Brunner, TU Dresden, showed applications of NMR techniques to characterize the structure, dynamics and flexibility of porous metal organic framework (MOF) materials with a particular emphasis on breathing MOFs. In this fascinating class of MOF materials the pores can collectively open or close, depending on the sample size and gas pressure. These structural changes are monitored by a combination of solid-state NMR and in situ high-pressure NMR techniques.

Mikael Akke, Lund University, illustrated the power of NMR relaxation methods, in combination with surface plasmon resonance, to the study of allostery within the ligand-binding domain of the glucocorticoid receptor by detecting conformational exchange of ^13^C backbone and methionine side chains. In addition, a method of relaxation rate constant measurement based on non-uniform sampling and accordion spectroscopy was shown to decrease the experimental time by an order of magnitude.

Christian Griesinger, MPI for Biophysical Chemistry, Göttingen, demonstrated methods to explore the kinetics of protein dynamics on both folded and unfolded proteins. Results from high-power (ca. 200 kHz) CPMG measurements were shown to measure life times of a few 100 ns. The talk also gave a glimpse of the potential of NMR in the analysis of minute amounts (10 s of micrograms) of natural compounds using deuterated and self-alignment in strong magnetic fields.

Isabella Felli, University of Florence, stressed on the potential of ^13^C detection and associated heteronuclear correlation experiments to reveal resonance assignments and subsequently structure and dynamics elucidation of a few IDP. Some of the dynamic and structural modules from these systems are not yet described in the PDB.

Anne Lesage, ENS Lyon, introduced the MAS-DNP method, which has become in recent years a valuable tool in solid-state NMR spectroscopy. In her talk, novel biradical polarizing agents (both dinitroxides and hybrid biradicals) were introduced, which provided excellent signal enhancements at high magnetic fields and high MAS frequencies. Using MAS-DNP, new information about catalytical materials and molecular substrates was obtained, which is otherwise inaccessible to NMR methods.

Malcolm Levitt, University of Southampton, presented studies of endofullerenes with NMR, THz spectroscopy and neutron scattering. The Southampton group has identified a number of unexpected NMR signals allowing them to determine for the first time, for instance, a J-coupling involving ^3^He and to probe NMR parameters of endofullerenes as a function of temperature and pressure. The phenomenon of ortho-para conversion in water molecules was studied by trapping them in the fullerene cage and generating strong non-thermal spin order.

The conference was organized by Konstantin Ivanov (ITC, Novosibirsk, Russia), Gerd Buntkowsky (TU, Darmstadt, Germany), Daniel Abergel (ENS Paris, France), and P. K. Madhu (TIFR Hyderabad, India). Suman Saurav, TIFR Hyderabad, provided technical assistance. The conference and seminar series were sponsored by Alexander von Humboldt Foundation, Wiley, Springer, HyperSpin, and Adani. Owing to the very favorable reactions and encouraging comments from the NMR community, there are already plans for ICONS 2021. For details and the schedule of upcoming talks, see the home page of the meeting ICONS2020^1^.

## Footnotes

1 Intercontinental NMR Seminar Series: “https://sites.google.com/view/nmr-seminar-series/home”

